# Dillapiole Dampens the Expression of the Major Virulence Genes of *Francisella tularensis*

**DOI:** 10.3390/molecules30193995

**Published:** 2025-10-06

**Authors:** Elliot M. Collins, Anthony Sako, Kristen Sikorsky, James Denvir, Jun Fan, Donald A. Primerano, Deanna M. Schmitt, Stuart Cantlay, Roger Seeber, Francisco León, Joseph Horzempa

**Affiliations:** 1 Department of Biological Sciences, West Liberty University, West Liberty, WV 26074, USA; emcollins@westliberty.edu (E.M.C.); anthony.sako@newhorizonsfoodsolutions.com (A.S.); kristen.sikorsky@usda.gov (K.S.); deanna.schmitt@westliberty.edu (D.M.S.); stuart.cantlay@westliberty.edu (S.C.); seeberrg@westliberty.edu (R.S.); 2 Department of Biomedical Sciences, Joan C. Edwards School of Medicine, Marshall University, Huntington, WV 25701, USA; denvir@marshall.edu (J.D.); primeran@marshall.edu (D.A.P.); 3 Texas A&M Institute for Genome Sciences and Societies, Texas A&M University, College Station, TX 77843, USA; fanj219@tamu.edu; 4 Department of Drug Discovery and Biomedical Sciences, College of Pharmacy, University of South Carolina, Columbia, SC 29208, USA; jleon@mailbox.sc.edu

**Keywords:** *Francisella tularensis*, Fennel, *Foeniculum vulgare*, dillapiole, 6-Allyl-4,5-dimethoxy-1,3-benzodioxole, pathogenesis, virulence, antibiotic, MglA, SspA

## Abstract

*Francisella tularensis* is a pathogenic bacterium and the causative agent of the disease tularemia. Because of the virulence of this bacterium and the potential for weaponization, the Centers for Disease Control and Prevention (CDC) has classified *F. tularensis* as a Category A Bioterrorism Agent. Therefore, the need for new treatments for tularemia is critical. In this work, we screened a cataloged library of natural extracts to identify those that inhibit the growth of *F. tularensis* only during infection of THP-1 monocyte cells. One of the most promising extracts identified in this screen was derived from *Foeniculum vulgare* (fennel). Using bioassay-guided fractionation, the fennel extract was fractionated, and the bioactive compound was isolated and structurally elucidated as the phenylpropanoid dillapiole. We subsequently confirmed that dillapiole alone could limit the replication of *F. tularensis* in infected THP-1 cells, but not outside of this infection model. Investigations on host responses suggested that dillapiole was not substantially augmenting the immunity of these THP-1 cells. We then investigated the potential virulence modulation activity of dillapiole. To test this hypothesis, RNA-seq analysis was carried out on *F. tularensis* bacteria that were treated with dillapiole. This showed that dillapiole caused a significant downregulation of genes controlled by the transcriptional regulators MglA and SspA, including those encoded in the *Francisella* pathogenicity island. Western blotting validated these findings as both IglA and IglC expression was diminished in *F. tularensis* LVS bacteria treated with dillapiole. Because dillapiole dampens the virulence gene expression of *F. tularensis*, we concluded that this compound has potential to be used as a novel therapeutic for tularemia with a unique mechanism of action.

## 1. Introduction

*Francisella tularensis* is one of the most virulent bacteria on the planet [1] and the causative agent of tularemia. This organism has an extremely low infectious dose (fewer than 10 colony-forming units [CFU]) and can cause a fatal disease if inhaled. Therefore, the Center for Disease Control and Prevention (CDC) has designated *F. tularensis* as a Category A Bioterrorism Agent (which is the category of greatest concern). Weaponization of multidrug-resistant *F. tularensis* could be catastrophic. There is no licensed vaccine for tularemia approved for human use, representing a substantial vulnerability to this select agent. Therefore, there is a need to develop novel therapeutics and strategies to reduce the morbidity and mortality caused by this pathogen.

The *Francisella* pathogenicity island (FPI) is a 30 kb region of the *F. tularensis* genome that is essential for intracellular growth and virulence in mice [2,3]. Virulent clades (Type A and Type B) of *F. tularensis* contain duplicate copies of the FPI, while the genome of *F. novicida*, another member of the *Francisella* genus, encodes only a single copy [4]. Within the FPI, there are 19 genes, including the intracellular growth locus (*iglA-J*), the pathogenicity determinant protein family (*pdpA-E*), *vsrG*, *dotU*, and *anmK* [2,4]. Most of these genes encode a non-canonical Type VI secretion system (T6SS) and its effectors [2,5]. The T6SS, which resembles a phage tail, is used to transport virulence factors across the host cell membrane [6]. The structural components of the T6SS are encoded by *iglA*, *iglB*, *iglC*, *iglD*, *pdpA*, and *pdpD*, with a complex of IglA and IglB forming the sheath, and the inner tube which is composed of IglC [6]. IglC is the best characterized FPI protein and plays roles in facilitating bacterial escape from the phagosome, NF-κB downregulation, and induction of apoptosis in macrophages [7,8,9]. The spike of the T6SS used to puncture host cell membranes is composed of VgrG and PdpA [6]. IglE functions to anchor the T6SS to the outer membrane, and this protein interacts with PdpB and DotU to form an inner membrane complex and periplasm-spanning channel [6]. AnmK is only expressed in *F. novicida* and is non-essential for intracellular growth [10]. The FPI genes *iglH*, *iglI*, and *iglJ* have been shown to be essential for replication in host cells, but their roles are still unknown [6].

There are multiple transcription factors that regulate the expression of the FPI genes. The main regulators of the FPI are MglA and SspA, global transcriptional regulators of over 100 genes in *F. tularensis* [11]. In order to regulate transcription, MglA forms a heterodimeric complex with SspA to associate with the RNA polymerase [12]. The orphan response regulator PmrA regulates the expression of genes inside and outside of the FPI, with few genes regulated by both PmrA and MglA [13,14]. Regulated by the MglA/SspA complex, FevR operates in tandem with MglA to increase the production of FPI proteins [14]. MigR is another virulence regulator for *F. tularensis*, regulating both FevR and the *iglABCD* operon [15]. Currently, Hfq is the only known negative regulator of the FPI and functions to downregulate the *pdp* operon [16]. A compound that would inhibit MglA/SspA-mediated transcriptional activation could serve as a novel therapeutic for *F. tularensis* infections.

Tularemia can be effectively treated with antibiotics if given early in infection [17,18]. Antibiotic treatment has been shown to decrease the mortality rate of tularemia from 30–60% to 2% when administered in time [19]. The three approved tularemia treatments include tetracyclines, and aminoglycosides [1,20]. While antibiotic treatments are effective, the symptoms of tularemia are non-specific, which can lead to misdiagnoses, resulting in delayed treatment and reduced survival [17]. While these treatments are effective in most cases, relapse and treatment failure are possible with tetracycline and gentamicin treatment [21]. Nephrotoxicity and ototoxicity are also concerns with aminoglycoside antibiotic treatments that need to be accounted for when treating tularemia [21]. There have been no documented naturally occurring cases of tularemia being resistant to these antibiotics. However, due to *F. tularensis’* bioterrorism potential and past biological weapon research, there is a potential for the release of antibiotic-resistant strains [19]. Due to these concerns, there is an interest in developing novel therapies for tularemia.

Natural products are a potential source for novel antimicrobial compounds. Natural products are compounds produced by plants, animals, fungi, and microorganisms that have potential medicinal applications [22,23]. Based on an analysis by the Food and Drug Administration (FDA), thirty-four percent of new medications approved between the years of 1982 and 2010 were isolated or derived from natural products. Medications isolated from natural products include statins, immunosuppressants, and anti-cancer drugs [22]. The majority of the antibiotics in use today are natural products, as they were isolated from or based on compounds from bacteria or fungi [22,24]. Although the number of synthetic compounds available is approximately 138 times higher than the number of natural products, the “hit rate” for natural products is higher; over 80% have been found to contain pharmacologically active compounds that can expedite the screening process [22].

*Foeniculum vulgare*, commonly known as fennel, is a biennial aromatic medicinal plant belonging to the family Apiaceae. A hardy herb with yellow flowers and feathery leaves, fennel is aromatic and flavorful, commonly used in food and traditional medicine [25]. The bulbs, foliage, and seeds are all edible, being used in many regional cuisines throughout the world. Originally from the Mediterranean coastal regions, fennel has been adopted by cultures across the globe, growing in dry soil close to shorelines and river banks [25]. In addition to the Mediterranean, fennel is popular in the Middle East and the Indian subcontinent. Fennel is also used medicinally as a carminative, diuretic, and a galactagogue [25]. With widespread use in traditional medicine, fennel has been the subject of multiple efforts to identify potential drug candidates.

The essential oil of fennel has been shown to contain a wide variety of phenols, flavonoids, and other biologically active compounds [25]. Some of the main compounds found in *F. vulgare* essential oil are anethole, caffeoylquinic acid, acacetin, and oleanolic acid [25]. There have been multiple studies to evaluate the antibacterial, antifungal, antidiabetic, anti-inflammatory, and antithrombotic properties of the essential oil and its components [25]. Although fennel essential oil has shown promise in many of these studies, not much progress has been made beyond the initial screen [25]. Estragole, a main component of fennel essential oil, is a known carcinogen, and it therefore poses a safety concern for the medicinal use of this oil [25]. However, since numerous screens have shown the therapeutic potential of the essential oil of fennel, additional studies should be conducted to identify bioactive molecules that could be developed into novel therapeutic treatments.

In this paper, we describe the major bioactive secondary metabolite isolated from fennel following a bioassay-guided fractionation methodology. We show that the bioactive compound, dillapiole, limits the intracellular growth of *F. tularensis* in THP-1 monocyte cells, likely by dampening the expression of major virulence genes within the FPI. Dillapiole and other natural products that diminish bacterial pathogenesis represent a novel paradigm for the treatment of bacterial infections.

## 2. Results

### 2.1. Natural Product Library Screen

**Figure 1:**
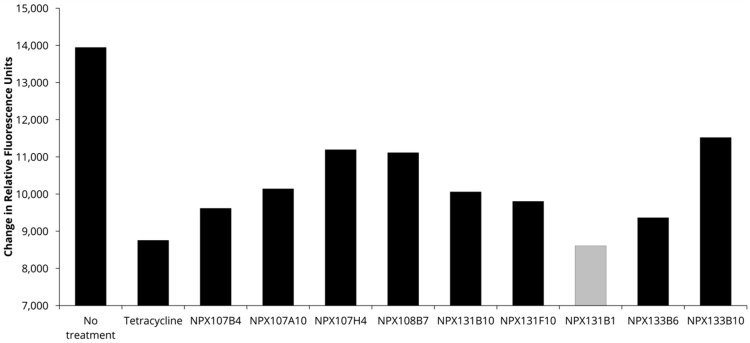
Extracts that inhibited *F. tularensis* LVS growth in an in vitro infection model. Extracts were considered to have inhibited growth if the change in bacterial fluorescence intensity (FI) was 5 standard deviations lower than the no-treatment control wells. The change in fluorescence intensity was calculated by taking the difference in the 0 h and 48 h time points. Shown are the 9 extracts that only inhibited growth in the THP-1 infection model, with one extract inhibiting growth better than the tetracycline positive control (NPX 131 B1, *F. vulgare*, fennel, gray bar).

A cataloged library of over 3200 extracts from plants, fungi, and marine life obtained from the National Center for Natural Products Research was screened to identify compounds that either increased innate immunity or diminished the pathogenesis of *F. tularensis*. The screen utilized red fluorescent *F. tularensis* LVS/pTC3D bacteria incubated with THP-1 monocytes, where changes in fluorescence intensity over time were used to measure intracellular bacterial replication. The initial screen identified 58 extracts that appeared to inhibit the growth of *F. tularensis* in the THP-1 infection model (unpublished data). Growth was considered to be inhibited if the change in fluorescence intensity for the extract was 5 standard deviations less than the fluorescence from the wells that were untreated. Extracts that inhibited LVS growth only during infection of THP-1 cells, but not in a disk diffusion assay nor during growth in broth culture (Table 1), were selected for further analysis. This step eliminated extracts acting as traditional antibiotics (i.e., compounds that interfere with an essential biological pathway of the bacteria). The results of this screen identified nine extracts (Table 1) that reduced bacterial replication in the THP-1 infection model (Figure 1) but did not impede bacterial growth in the absence of host cells. Given these data, those nine extracts potentially contained compounds that diminished the virulence of *F. tularensis* or augmented the immune response.

### 2.2. Screen Validation

**Figure 2:**
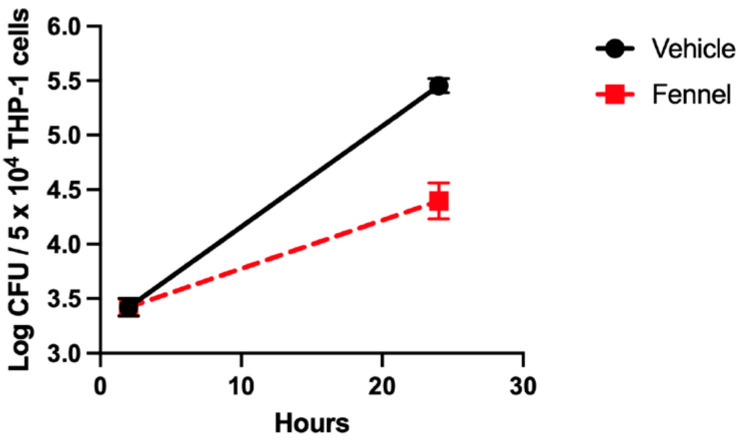
Extracts reduced *F. tularensis* LVS intracellular replication. *F. tularensis* LVS was used to infect THP-1 cells, and after a two-hour incubation for the bacteria to invade the THP-1 cells, gentamicin was administered to kill any remaining extracellular bacteria. Gentamicin and dead bacteria were removed with a wash, and the cells were treated with either vehicle or fennel extract. Lysates from 2 and 24 h post infection were used to enumerate CFUs of intracellular *F. tularensis* LVS present. The fennel (*F. vulgare*) extract showed significant inhibition of bacterial growth compared to the control. *p* ≤ 0.0001. Data represent mean CFU ± SD.

To validate the results of the initial screen, a gentamicin protection assay was employed to enumerate the number of intracellular *F. tularensis* within the THP-1 cells following treatment with selected extracts (Table 1). The *Foeniculum vulgare* (fennel) root extract of the original nine showed the highest inhibition of growth in the initial screen and was selected for validation in a separate bacterial intracellular growth assay (Figure 2). As expected, fennel extract in this validation experiment significantly diminished the growth of *F. tularensis* LVS within the THP-1 cells (Figure 2). These data confirmed the findings of the screen and compelled us to move forward with bioassay-guided fractionation to identify the novel antimicrobial or immune-augmenting compounds.

### 2.3. Bioassay-Guided Fractionation

**Figure 3:**
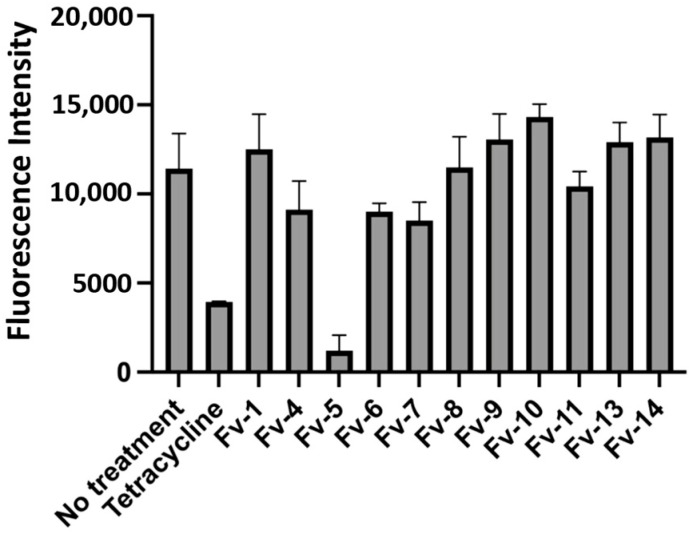
Fraction Fv-5 from *F. vulgare* decreases bacterial replication. From the fourteen fractions produced from the *F. vulgare* extract, only one showed a decrease in the growth of *F. tularensis* LVS/pTC3D over a 48 h period. The results indicated that the active compound eliciting the novel antimicrobial or immune-augmenting effect was present in the Fv-5 fraction. TLC showed that the fraction was a mixture of compounds, which required further separation to identify the active compound. Data represent mean FI ± SD.

**Figure 4:**
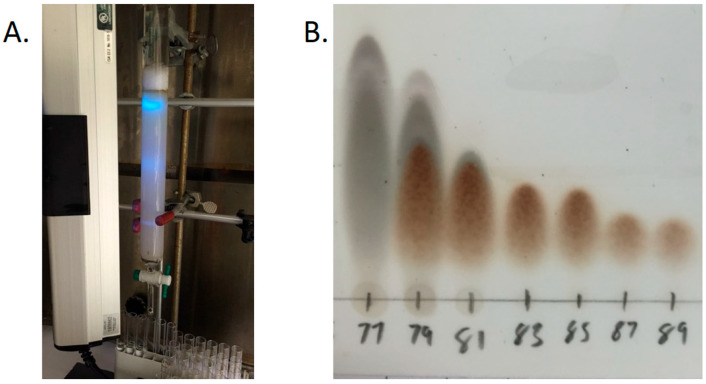
Subfractionation of Fv-5. (**A**)**.** Column from the subfractionation of Fv-5 exposed to short-wave UV light. The fluorescent bands are produced by conjugated compounds with Pi-bonding electrons. The central diffuse band was later identified as the active compound from Fv-5. (**B**)**.** TLC analysis of Fv-5 subfractions. Silica TLC plates were stained with vanillin-H_2_SO_4_ to visualize the compounds present under visible light. Based on these results, the 182 original fractions were combined into 13 main subfractions based on their purity. In this image, fractions 83–89 appeared to contain the same pure compound, while fraction 79 appears to contain a mixture of at least four compounds.

Following the validation of the efficacy of the extracts to inhibit the growth of *F. tularensis* LVS in infected THP-1 cells, we began to isolate and identify the active compounds within the *F. vulgare* extract. To accomplish this, we utilized normal-phase column chromatography to separate all the compounds within this extract by polarity. The *F. vulgare* extract was separated into fourteen fractions, all of which were subsequently tested for inhibition of the red fluorescent strain of *F. tularensis* LVS during infection of THP-1 cells. We identified one fraction, Fv-5, that produced a substantial decrease in fluorescence (a surrogate for intracellular growth) compared to the no-treatment control (Figure 3). Additionally, this decrease in fluorescence was comparable to treatment with the clinically used antibiotic tetracycline (Figure 3). Given these data, the Fv-5 fraction was selected for further separation as this fraction contained multiple different compounds of similar polarity (Figure 4).

**Figure 5:**
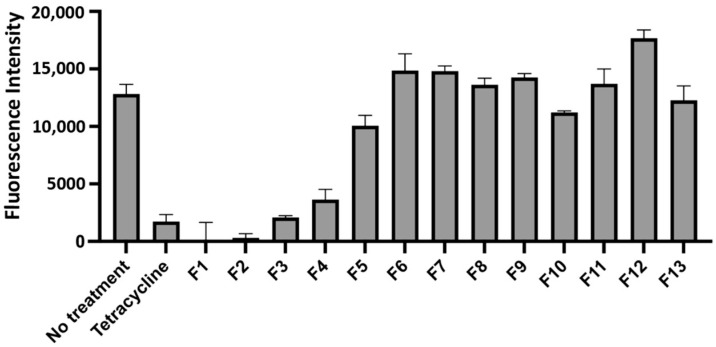
Subfractions of Fv-5 showed reduced bacterial replication. Thirteen subfractions were made from Fv-5 following column chromatography. Four of the thirteen showed inhibited growth of *F. tularensis* LVS/pTC3D, with two showing a larger decrease in replication than the tetracycline positive control. These data allowed us to move onto the identification of the active compound, eliciting either an immunostimulatory or virulence-dampening effect. Data represent mean FI ± SD.

To achieve separation, fraction Fv-5 was subjected to normal-phase column chromatography (Figure 4A) with smaller step changes in solvent polarity. In total, 182 fractions were collected and then combined into thirteen subfractions based on the composition revealed by TLC (Figure 4B). These thirteen subfractions were tested for activity with the same screen as all previous fractions, with the results indicating that four of the thirteen subfractions showed a large decrease in *F. tularensis* replication as measured by a reduction in fluorescence intensity (Figure 5). Based on these data and the TLC, subfraction 2 was selected for compound identification as this fraction consisted of a pure compound capable of inhibiting bacterial replication within THP-1 cells.

### 2.4. Compound Identification

The purified bioactive compound from subfraction 2 of fraction Fv-5 was subjected to nuclear magnetic resonance (NMR) for identification. The proton ^1^H-NMR spectrum in deuterated benzene showed the presence of a single aromatic proton at δ 6.41 ppm, vinylic protons at δ 5.91 (1H) and 5.00 ppm (2H), CH_2_ adjacent to the aromatic ring, and the vinyl group at 3.32 ppm (2H). A COSY experiment allowed us to the see the coupling system, where the benzylic CH_2_ at 3.32 (*d*, *J* = 6.6 Hz, 2H) ppm has a clear correlation with the vinylic proton at 5.91 (*ddt*, *J* = 16.7, 10.0, and 6.6 Hz, 1H) ppm, and the last showed a correlation with the exomethylene protons at 5.02 (*dq*, *J* = 17.0, 1.7 Hz, 1H), and 4.98 (*dq*, *J* = 10.0, 1.4 Hz, 1H) for the aliphatic chain in the molecule. The presence of the typical CH_2_ cyclic ketal (acetonide) on the aromatic ring is found as a singlet at δ 5.25 ppm (2H), and the last signals are the methoxy groups at δ 3.76 (3H) and 3.64 ppm (3H) (see Supplementary Materials). The carbon NMR and 2D NMR (COSY, HSQC, HMBC) support the chemical structure proposed [26]. After NMR spectra were obtained, gas chromatography mass spectrometry (GC/MS) and low-resolution electrospray ionization mass spectrometry (ESIMS) were performed to determine a molecular mass for the unknown compound. This revealed 223 *m*/*z* [M + H]^+^ from the mass spectrometry data and the molecular formula of C_12_H_14_O for the unknown compound. These data coupled along with the NMR data allowed us to elucidate that the unknown active compound was dillapiole (see Figures S1–S8 in the Supplementary Materials).

### 2.5. Minimum Inhibitory Concentration (MIC)

**Figure 6:**
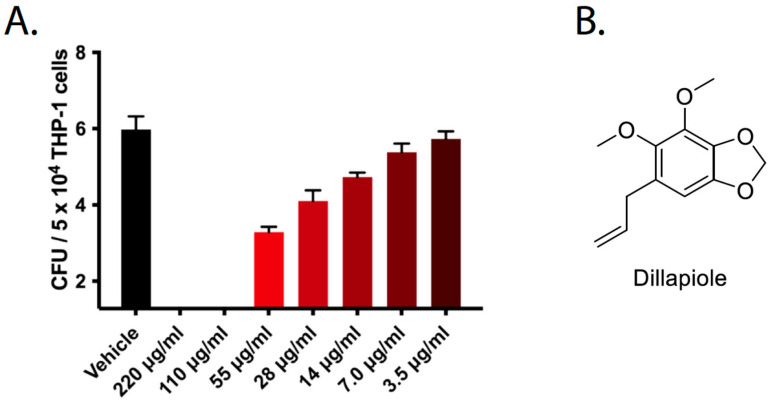
*F. tularensis* growth in THP-1 cells was inhibited by dillapiole. (**A**) THP-1 cells were infected with *F. tularensis* LVS. After a two-hour incubation, cells were treated with gentamicin to kill any remaining extracellular bacteria. Infected cells were then treated with the indicated concentrations of dillapiole, and after 22 h, cells were lysed and bacteria were enumerated. Data represent mean CFU ± SD. *p* < 0.0001 for 14–220 μg/mL vs. vehicle; *p* < 0.05 for 7 μg/mL vs. vehicle; 3.5 μg/mL vs. vehicle was not significantly different as determined by a one-way ANOVA with Dunnett’s multiple comparisons test. (**B**) The structure of dillapiole.

After dillapiole was identified as the active compound from the *F. vulgare* extract, we sought to verify whether this compound alone was responsible for the decrease in *F. tularensis* LVS replication in infected THP-1 cells. Additionally, we wanted to determine the MIC of dillapiole to see if this compound exhibited activity within the desired therapeutic range. To validate the finding that the extracted dillapiole was the active compound, we used commercially available dillapiole from Sigma Aldrich and tested its activity in a gentamicin protection assay. Commercial dillapiole inhibited the growth of *F. tularensis* LVS in THP-1 cells with an MIC of less than 7 μg/mL (Figure 6). During this assay, the THP-1 cells were inspected microscopically to evaluate whether dillapiole was toxic to mammalian cells. No change in THP-1 cell morphology or integrity was observed at the tested concentrations (unpublished data). Additional LDH release assays indicated that dillapiole did not induce the death of mammalian cells at the concentrations tested here (unpublished data).

### 2.6. Bacterial Clearance by Dillapiole Is Not Due to a Clear Augmentation of the Host Response

**Figure 7:**
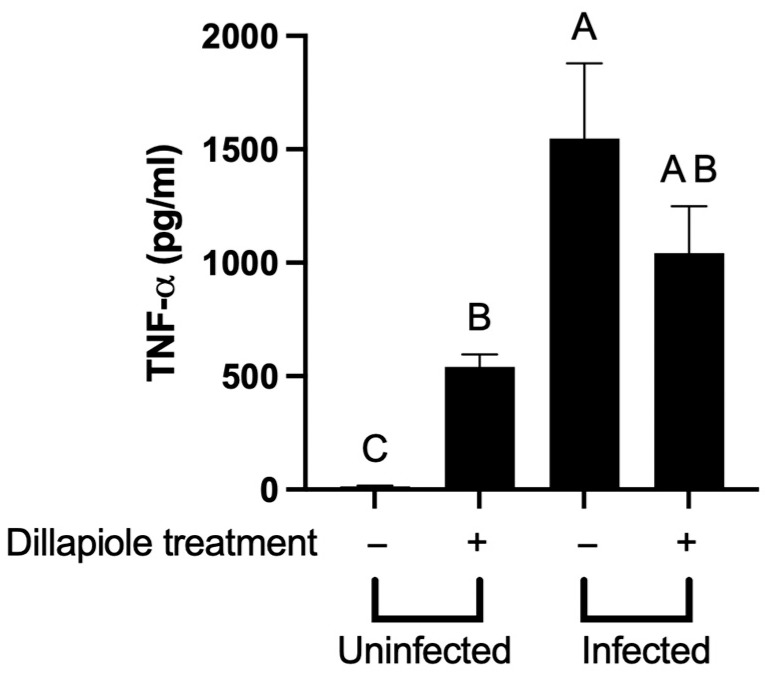
TNF-α concentration in THP-1 cells treated with dillapiole. THP-1 cells were either infected with *F. tularensis* LVS or left uninfected. Two hours post infection, both an infected and uninfected group were treated with dillapiole. After a 24 h incubation, the supernatants were collected, and an ELISA was performed to determine the TNF-α concentration of the test groups. Data were analyzed with a one-way ANOVA with Tukey’s multiple means comparison test. Letters above each column represent statistically significant categories (*p* < 0.05).

We next sought to determine the mechanism of action of dillapiole. Using a co-culture system, Eneslätt et al. showed that peripheral blood mononuclear cells (PBMCs) infected with *F. tularensis* that were co-cultured with non-adherent PBMCs from immunized individuals could control the intracellular growth of these bacteria [27]. Expression of IFN-γ, TNF, and MIP-1β correlated with the immune-mediated bacterial control and could have played a role in diminishing infection [27]. We reasoned that if dillapiole was mediating the clearance of *F. tularensis* in THP-1 cells by augmenting immunity, we would see a similar response to the observations of Eneslätt et al. To do so, we analyzed the global transcriptional changes in THP-1 cells treated with dillapiole utilizing RNA-seq. This analysis revealed that the expression of 1174 genes of the THP-1 cells was significantly affected by dillapiole differently in the infected cells to in the uninfected cells. Gene expression changes were further analyzed by Ingenuity Pathway Analysis (IPA), which indicated that 45 THP-1 biological pathways were affected by dillapiole (Figure S9 in the Supplementary Materials). Here, we observed that the expression of the TNF-receptor pathway was diminished by dillapiole while neither the IFN-γ nor the MIP-1β signaling pathways were affected by dillapiole (Figure S9). While the RNA-seq analysis indicated that the expression of the TNF receptor pathway was diminished, we observed increased TNF-α protein production by uninfected THP-1 cells that were treated with dillapiole compared to cells that were untreated (Figure 7). However, no significant difference in TNF-α levels was observed between infected cells treated with or without dillapiole (Figure 7). Regardless, increased production of TNF-α protein alone is unlikely to be responsible for the diminished intracellular replication of *F. tularensis* LVS as others have shown that TNF-α treatment did not reduce intracellular replication of *F. tularensis* [28].

In addition to THP-1 cells, we used the RAW 267.4 murine macrophage line to study the effect of dillapiole on host immunity and the pathogenesis of *F. tularensis* [29,30]. To determine if dillapiole could diminish bacterial replication in these mouse cells, RAW 267.4 macrophages were infected with the red fluorescent *F. tularensis* LVS/pTC3D, treated with dillapiole, and fluorescence was measured as a surrogate for bacterial replication (Figure S10 in the Supplementary Materials). As in the THP-1 cells, dillapiole was sufficient for limiting bacterial replication in these RAW 264.7 cells (Figure S10 in the Supplementary Materials). To determine the impact on host immunity, we sought to analyze the production of over 20 different proinflammatory cytokines by RAW 267.4 murine macrophages in response to dillapiole using the Bio-Rad BioPlex system. Surprisingly, the only cytokine significantly affected by dillapiole was TNF-α (Figure S11 in the Supplementary Materials). In contrast to the observation made in THP-1 cells, treatment with dillapiole led to a reduction in the production of TNF-α protein by the RAW 267.4 cells; this result was confirmed by ELISA (Figures S11 and S12 in the Supplementary Materials). This decrease in TNF-α alone is unlikely to be responsible for the diminished intracellular replication of *F. tularensis* LVS in the RAW 264.7 macrophage cells [31]. This result suggests that the disparate TNF-α production observed from the RAW 267.4 cells compared to the THP-1 cells was either unrelated to bacterial control or due to species-specific immune responses. Furthermore, the cytokine data indicates that dillapiole may be having a more profound effect on bacterial physiology rather than the host response.

### 2.7. Dillapiole Diminishes F. tularensis Virulence Gene Expression

To investigate the possibility that dillapiole acts as a virulence modulator, we utilized RNA-seq to determine the difference in gene expression between *F. tularensis* LVS treated with and without dillapiole. Following treatment with dillapiole, 160 genes showed a significant decrease in expression compared to the vehicle control group. Included in these 160 genes were genes present on the *Francisella* pathogenicity island (FPI) controlled by MglA and SspA (Table 2). Additionally, treatment with dillapiole resulted in a significant decrease in expression of the transcriptional regulator SspA (*p*-value 0.017). Since these transcriptional activators are required for pathogenesis and intracellular growth of *F. tularensis*, we conclude that dillapiole attenuates the pathogenesis of *F. tularensis* by inhibiting the activity of MglA and SspA by the direct decrease in SspA transcription.

**Figure 8:**
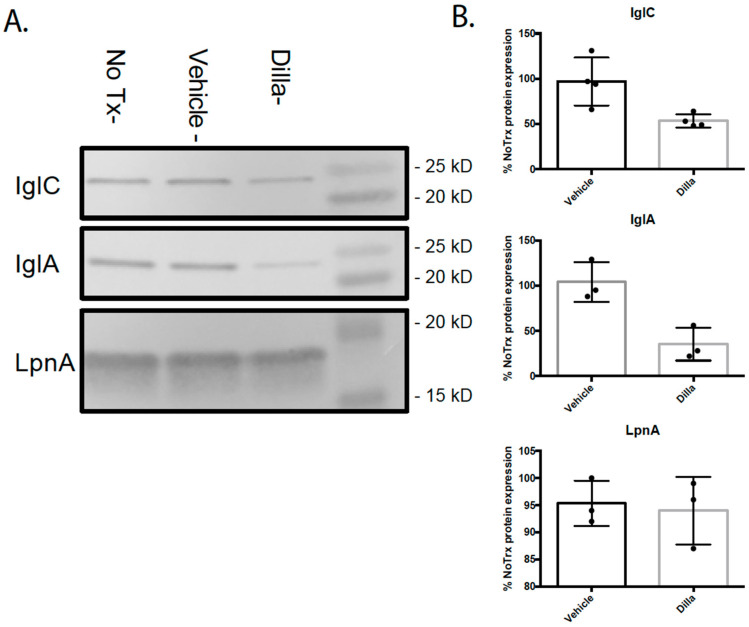
Dillapiole treatment reduces IglA and IglC protein expression in *F. tularensis* LVS. (**A**) Levels of IglC, IglA, and LpnA were detected by Western blot analysis with or without dillapiole treatment. Digital images of the blots were analyzed using ImageJ, and data generated from 3 to 4 independent replicate blots were analyzed using a paired t test (% no treatment protein expression of vehicle or dillapiole-treated bacteria). (**B**) Graphs of quantified protein levels created using ImageJ. For dillapiole vs. vehicle, IglC *p* = 0.0305 and IglA *p* = 0.0040; for LpnA, *p* = 0.7157.

To confirm whether the decrease in FPI gene transcription following dillapiole treatment led to a decrease in protein levels, we conducted Western blotting on lysates from *F. tularensis* LVS cells treated with dillapiole or vehicle. We assessed IglC and IglA levels as they are encoded for on the FPI, and LpnA, an outer membrane protein produced by *F. tularensis*, whose transcription was not shown to be affected by treatment with dillapiole. The Western blots showed a significant decrease (~2-fold) in IglC and IglA production in the cultures treated with dillapiole, while the protein levels of LpnA were unaffected by treatment (Figure 8A,B). These data are consistent with the RNA-seq results.

## 3. Discussion

While development of novel antimicrobial compounds has slowed, the threat of antibiotic resistance continues to grow as pathogenic bacteria continually evolve. The aim of this work was to approach this problem from a new perspective, looking for novel compounds that would augment the innate host immune system or dampen the virulence of the pathogenic bacteria. Our screen of a cataloged natural product library identified nine extracts that showed novel antimicrobial action with the inhibition of bacterial growth in an infection model, but no direct killing of these bacteria as is the case with traditional antibiotics. Following the identification of these candidate extracts, we identified dillapiole as the active compound that limited the growth of intracellular *F. tularensis* from the *F. vulgare* (fennel) extract.

After dillapiole was identified as the active compound, our investigation shifted to finding the mechanism of action causing the reduction in growth. Our first hypothesis was that dillapiole was acting as an immunomodulator. To test for this possibility, we performed a multiplex cytokine assay, ELISA, and RNA-seq analyses to look at the changes in the immune cells treated with dillapiole. No clear augmentation of host immunity was observed between treatment and control groups that could have been responsible for the observed reduction in intracellular bacteria. However, future investigation into host pathways affected by dillapiole could provide additional insight into the mechanism of this compound. Identification of naturally occurring plant material that augments protective host immunity is not unprecedented. For instance, in a mouse model of tularemia, nasal administration of polysaccharides from Acai enhanced the production of IFN-γ by lung NK cells to levels that were both protective and therapeutic [32].

The second possibility was that dillapiole was affecting the expression of bacterial virulence genes. RNA-seq was used to look for global transcriptional changes in *F. tularensis* when treated with dillapiole. The resulting data showed that dillapiole downregulates the expression of FPI genes and the SspA gene; this suggests that dillapiole attenuates the *F. tularensis* pathogenesis by impeding the activity or formation of the MglA/SspA regulatory complex by limiting SspA. Western blotting confirmed that FPI-encoded proteins exhibited diminished expression in the presence of dillapiole, which is consistent with the RNA-seq data.

Several small molecules are known to modulate bacterial virulence including SE-1, which inhibits the virulence factor regulator VirF in *Shigella flexneri*, leading to a decrease in AraC family proteins needed for pathogenesis [33]. Another small molecule, M21, reduces the expression of multiple virulence factors in methicillin-resistant *Staphylococcus aureus*, returning it to a non-virulent state [34]. These virulence-regulating molecules could play a key role in the development of novel treatments for the threat of antibiotic resistance to human health. Dillapiole could potentially act by blocking the binding of guanosine tetraphosphate (ppGpp) to the MglA/SspA complex, which is needed to activate full FPI transcription. MglA/SspA trans-activation functionality could be tested utilizing PigR response element reporter assays [35,36].

Dillapiole is a phenylpropanoid most commonly found in high concentrations in the essential oil of *Piper aduncum* [23,30,31]. With the relative ease of extracting dillapiole from *P. aduncum* essential oil, it has been the focus of many studies to identify potential medical uses [23,30,31,32]. Dillapiole was also found as the major volatile compound in a sample of extract of *F. vulgare* collected in the Balkans region in Europe [33]. The anti-inflammatory effects of dillapiole were evaluated using a rat paw edema model to compare dillapiole and synthesized analogs against indomethacin; however, none of the dillapiole analogs were shown to be as effective as the reference drug [30]. Dillapiole has also been investigated as a potential treatment for leishmaniasis [32]. Data from the in vitro studies show that dillapiole is mildly active as an antileishmanial, but falls far short of the effectiveness of the reference drug, amphotericin B [32]. Another study looked at the potential use of dillapiole to control tick populations. It has been shown to exhibit acaricidal effects against *Amblyomma sculptum* larvae [31]. While dillapiole has been studied for numerous different uses, the novel antimicrobial effects we have demonstrated have not been previously documented.

Future studies should be conducted to explore the potential of dillapiole as a pharmaceutical. These include toxicity studies, pharmacokinetic/pharmacodynamic investigations, and assessment of the therapeutic efficacy of dillapiole in a murine model of tularemia. This work will better assess the effects of plasma protein binding, metabolization, bioavailability, and excretion on the activity of dillapiole. Additional studies should explore whether dillapiole shows efficacy against fully virulent *F. tularensis* and other bacterial species.

In summary, dillapiole is a novel antibacterial therapeutic that exerts its activity by dampening virulence factor expression in the pathogenic bacterium *F. tularensis* and potentially others. This treatment paradigm is different from that of existing antibiotics and therefore minimizes the selection for antibiotic resistance. This is because traditional antibiotics target central biological processes that are required for normal viability and growth of the bacteria (even outside the context of infection) while dillapiole only affects the expression of genes required for pathogenesis and does not affect bacterial viability. Therefore, dillapiole does not exert the same degree of selective pressure as traditional antibiotics, which will likely minimize the development of resistance to this novel treatment. Addition of virulence modulators to our expanding repertoire of treatments for infections could lead to a reduction in the morbidity and mortality resulting from antibiotic-resistant infections in the future.

## 4. Materials and Methods

### 4.1. Bacterial Culture Conditions for F. tularensis

LVS was provided by Dr. Karen Elkins (U.S. Food and Drug Administration, Silver Spring, MD, USA). Bacteria that were stored as freezer stocks were used to inoculate chocolate II agar plates that were at 37 °C with 5% CO_2_ for three days. Bacteria from agar plates were used to inoculate sterile filtered TSBc broth [Tryptic Soy Broth (BD, Franklin Lakes, NJ, USA) supplemented with 0.1% L-Cysteine hydrochloride (Fisher, Waltham, MA, USA)]; these bacteria were then grown at 37 °C with shaking for 14–18 h. For *F. tularensis* LVS/pTC3D, kanamycin (10 μg/mL) was added to the media.

### 4.2. Natural Product Library Screen

Through a collaboration with the National Center for Natural Products Research, a cataloged library of over 3200 extracts from plants, fungi, and marine life was screened for proinflammatory or anti-pathogenesis activity using a THP-1 infection model [37]. THP-1 cells (ATCC TIB-202) were cultured in RPMI 1640 medium (no phenol red) supplemented with 10% fetal bovine serum (Gemini Bio-Products, Sacramento, CA, USA), 25 mM HEPES (Corning, Glendale, AZ, USA), 1 mM sodium pyruvate (Gibco, Waltham, MA, USA), and 1% glutamine dipeptide (Gibco). THP-1 cells were plated in flat-bottomed 96-well plates at a density of 5 × 10^4^ cells/well. *F. tularensis* LVS/pTC3D cultures were adjusted to an OD_600_ of 0.3 (~1 × 10^9^ CFU/mL) and diluted to a multiplicity of infection (MOI) of 10 in pre-warmed THP-1 media supplemented with kanamycin (10 μg/mL) to ensure the selection of the plasmids harbored by these bacteria. Wells were treated with an extract from the NPX library (8 μg/mL) or tetracycline (20 μg/mL). The plates were incubated at 37 °C, 5% CO_2_ for 48 h. Fluorescence readings were taken on a microplate reader (BioTek, Winooski, VT, USA) using an excitation wavelength of 435 nm and an emission wavelength of 495 nm at 0 h, 24 h, and 48 h time points.

### 4.3. Kirby–Bauer Disk Diffusion Assay

Kirby–Bauer disk diffusion assays were performed on chocolate II agar plates using extracts previously identified to inhibit the replication of *F. tularensis* in high-throughput testing. *F. tularensis* cultures were adjusted to an OD_600_ of 0.1, and 100 μL of this diluted culture was spread and plated onto each chocolate II agar plate. Four sterile Whatman filter disks were placed on each plate, and 5 µL of the extracts, gentamicin (50 mg/mL) or phosphate-buffered saline (PBS), was aliquoted onto each disk. Plates were incubated at 37 °C, 5% CO_2_ for 48 h. Zones of inhibition were measured in millimeters on perpendicular axes, and these values were averaged.

### 4.4. Gentamicin Protection Assay

THP-1 cells (ATCC TIB-202) were cultured in RPMI 1640 medium supplemented with 10% fetal bovine serum (Gemini Bio-Products), 25 mM HEPES (Corning), 1 mM sodium pyruvate (Gibco), and 1% glutamine dipeptide (Gibco). THP-1 cells were plated in flat-bottomed 96-well plates at a density of 5 × 10^4^ cells/well. *F. tularensis* cultures were adjusted to an OD_600_ of 0.3 (~1 × 10^9^ CFU/mL) and diluted to a multiplicity of infection (MOI) of 10 in pre-warmed THP-1 medium. The THP-1 cells were then exposed to *F. tularensis* for 3 h at 37 °C, 5% CO_2_. Subsequently, cells were incubated with Hank’s Balanced Salt Solution (HBSS) (Corning) containing gentamicin (100 μg/mL) for 60 min to kill extracellular bacteria. The THP-1 cells were then washed twice with HBSS after cells were pelleted at 100× *g* for 5 min. Following washes, fresh tissue culture medium was then added, and natural products (8 μg/mL) or dillapiole was added. Afterwards, infected THP-1 cells were incubated for an additional 20 h. To detect viable *F. tularensis* bacteria, at each time point indicated, THP-1 cells were lysed with 0.02% sodium dodecyl sulfate. These lysates were serially diluted and plated onto chocolate II agar plates. Plates were incubated at 37 °C, 5% CO_2_ for three days to enumerate *F. tularensis* CFU.

### 4.5. General Chemistry Procedures

One- and two-dimensional experiments were recorded on Bruker model AMX 400 and 500 NMR spectrometers operating with standard pulse sequences. The instruments were operating at 400 and 500 in ^1^H and 100 and 125 in ^13^C, respectively. CDCl_3_ and C_6_D_6_ were used as solvents, and TMS was used as an internal standard. Low-resolution mass spectra (MSs) were obtained on a Micromass Q-Tof Micro mass spectrometer with a lock spray source, as well as by gas chromatography–mass spectrometry (PerkinElmer Clarus SQ 8). Column chromatography was performed on silica gel (70–230 mesh, Merck). Fractions collected from column chromatography were checked by TLC (silica gel 60 F_254_), while preparative TLC was conducted on silica gel 60 PF_254+366_ plates (20 × 20 cm, 1 mm thick). Detection of compounds was carried out under UV light.

### 4.6. Fractionation

The dried ethanolic extract of *F. vulgare* was received from the National Center for Natural Product Research at the University of Mississippi. The extract was then dissolved in methanol (MeOH; 250 mL), and silica gel (50 g) was added. The mixture was dried under vacuum. The dried extract supported in silica was then added to a prepared silica gel column, eluted initially with 100% hexanes and then gradient eluted with hexanes: ethyl acetate (EtOAc) at ratios of 80:20, 60:40, 40:60, and 20:80, followed by 100% EtOAc. The 100% EtOAc elution was followed by another gradient solvent system with EtOAc: MeOH at ratios of 90:10 × 2 and 50:50 × 2, followed by 100% MeOH. The fractions were collected in 500 mL increments and monitored by TLC on silica gel using hexanes: EtOAc (80:20). Fractions were concentrated under vacuum to yield fourteen main fractions (Fv-1 to Fv-14). As fraction Fv-5 was determined to contain the active compound, Fv-5 was subjected to an additional round of fractionation using a silica gel column prepared with 100% hexanes. The column was initially eluted with 100% hexanes and then gradient eluted with hexanes: EtOAc at ratios of 99:1, 97:3, 10:90, 80:20, and 60:40, and finally, 100% MeOH. Subfractions were collected in 13 × 100 mm tubes and were changed once each tube was halfway full (~4 mL). Each subfraction was monitored by TLC on silica gel using hexanes (100%), EtOAc: hexanes (5:95), and EtOAc: hexanes (10:90). Similar subfractions were mixed and dried under vacuum to produce thirteen main subfractions (I to XIII).

### 4.7. Cytokine and Chemokine Assays

TNF-α levels in THP-1 cell supernatants were determined using the Quantikine ELISA Human TNF-α Immunoassay (R&D Systems, Minneapolis, MN, USA) on a microplate reader (BioTek) using the manufacturer’s protocol. To assess the immune response to dillapiole in murine cells, a RAW 264.7 infection model was used. RAW 264.7 cells (ATCC TIB-71) were cultured in Dulbecco’s modified Eagle’s medium (DMEM) supplemented with 10% fetal bovine serum (Gemini Bio-Products), 25 mM HEPES (Corning), 1 mM sodium pyruvate (Gibco), and 1% glutamine dipeptide (Gibco). RAW 264.7 cells were seeded in Primaria 96-well plates 24 h prior to infection at a density of 5 × 10^4^ cells/well. *F. tularensis* cultures were adjusted to an OD_600_ of 0.3 (~1 × 10^9^ CFU/mL) and diluted to a multiplicity of infection (MOI) of 10 in pre-warmed RAW 264.7 media. Infected RAW 264.7 cells were treated with dillapiole (8 μg/mL) or the vehicle (100% hexanes). The plates were incubated at 37 °C, 5% CO_2_ for 24 h followed by the collection of supernatants. Using a Bio-Plex 200 System (Bio-Rad), the Bio-Plex Pro Mouse Cytokine Th1/Th2 Assay (Bio-Rad) was used to determine chemokine and cytokine levels in RAW 264.7 cell supernatants. Bio-Plex Manager 6.0 software (Bio-Rad) was used to calculate cytokine and chemokine concentrations against a standard.

### 4.8. THP-1 RNA Extraction

THP-1 cells (ATCC TIB-202) were cultured in RPMI 1640 medium supplemented with 10% fetal bovine serum (Gemini Bio-Products), 25 mM HEPES (Corning), 1 mM sodium pyruvate (Gibco), and 1% glutamine dipeptide (Gibco). THP-1 cells were plated in flat-bottomed 6-well plates at a density of 9 × 10^6^ cells/well. *F. tularensis* cultures were adjusted to an OD_600_ of 0.3 (~1 × 10^9^ CFU/mL) and diluted to a multiplicity of infection (MOI) of 10 in pre-warmed THP-1 media. Uninfected and infected THP-1 cells were then treated with dillapiole (8 μg/mL) or hexane 1 h post infection. Plates were incubated for 24 h at 37 °C, 5% CO_2_.

After incubation, cells were transferred to 15 mL conical tubes and collected by centrifugation. After removing the supernatant, the pellets were resuspended in Trizol and incubated at room temperature for 5 min. Chloroform was then added and vigorously mixed for 15 s until the sample turned milky pink. The mixture was then transferred to an organic matrix tube and centrifuged at max speed for 15 min. After centrifugation, the clear liquid layer was transferred to a new tube, and 70% ethanol was added at a 1:1 ratio. The resulting mixture was then transferred to a spin column (Invitrogen PureLink™ RNA Mini Kit) and spun at max speed for 15 s. The flow through was discarded, and PureLink Dnase (Invitrogen, Waltham, MA, USA) was added directly to the column and incubated for 15 min at room temperature. Following Dnase treatment, Wash Buffer 1 was added to the columns and centrifuged for 15 s. The columns were then washed with Wash Buffer 2 twice, each time being spun for 15 s followed by a final 2 min spin to ensure the column was dry. Spin columns were transferred to recovery tubes, and total RNA was eluted with Rnase-free water. The columns were incubated at room temperature for 2 min before centrifuging for 1 min. RNA concentrations were determined using a NanoDrop spectrophotometer and then stored at −80 °C.

For RNA-Seq analysis of THP-1 cells (±dillapiole), total RNA was extracted from five replicate samples in each experimental group. RNA was quantitated with Qubit RNA fluorimetric assays (ThermoFisher Scientific, Waltham, MA, USA). RNA Integrity Numbers (RINs), assessed using 2100 Bioanalyzer RNA microfluidic chips, were greater than 8.5 for all samples. Illumina-compatible libraries were prepared using a TruSeq Stranded mRNA kit (Illumina, San Diego, CA, USA). RNA libraries were sequenced on an Illumina HiSeq1500 sequencer.

Sequencing reads were trimmed to remove Illumina adapter sequences and low-confidence base calls using Trimmomatic version 0.32 [38]. The quality of the resulting trimmed reads was verified using FastQC version 0.11.7 [39]. Reads were aligned to the reference human genome GRCh38 using HISAT2 version 2.1.0. Counts of reads mapping to known genes for each sample were carried out using the R/Bioconductor package GenomicAlignements, version 1.16.10.

Gene expression differences in THP-1 cells were computed using DESeq2 version 1.20.0. Specifically, the expression ratios of treated uninfected cells to untreated uninfected cells and of treated infected cells to untreated infected cells were computed, and then these ratios were compared by using a statistical model of expression as a function of infection, treatment, and their interaction. Genes with a significant interaction effect were identified. A threshold of a Benjamini–Hochberg adjusted *p*-value less that 0.1 was used to determine significance.

Resulting significant genes were analyzed using the Ingenuity Pathway Analysis tool to identify genetic pathways perturbed by dillapiole treated differently between the infected and uninfected cells.

### 4.9. F. tularensis RNA Extraction

Broth cultures of *F. tularensis* LVS treated with vehicle, dillapiole (8 μg/mL), or no treatment were grown to the stationary phase and normalized to the lowest OD_600_ and pelleted. After removing the supernatant, the pellets were resuspended in Trizol and incubated at room temperature for 5 min. Chloroform was then added and vigorously mixed for 15 s until the sample turned milky pink. The mixture was then transferred to an organic matrix tube and centrifuged at max speed for 15 min. After centrifugation, the aqueous phase was transferred to a new tube, and 70% ethanol was added at a 1:1 ratio. The resulting mixture was then transferred to a spin column (Invitrogen PureLink™ RNA Mini Kit) and spun at max speed for 15 s. After the flow through was discarded, PureLink Dnase (Invitrogen) was added directly to the column and incubated for 15 min at room temperature. Following Dnase treatment, Wash Buffer 1 was added to the columns and centrifuged for 15 s. The columns were then washed with Wash Buffer 2 twice; for each wash, the columns were centrifuged for 15 s followed by a final 2 min spin to ensure the column was dry. Spin columns were transferred to recovery tubes, and Rnase-free water was added to elute RNA. The columns were incubated at room temperature for 2 min before centrifuging for 1 min. The RNA concentrations were determined using a NanoDrop spectrophotometer and then stored at −80 °C.

### 4.10. F. tularensis RNA-Seq

For RNA-Seq analysis of *Francisella* cells (hexane vs. dillapiole-treated), total RNA was extracted from eight replicate samples in each experimental group. RNA Integrity Number (RIN) values as determined by Bioanalyzer electrophoresis were all above 9. RNA samples (2 μg) were dissolved in 15 μL TE buffer (10 mM Tris HCL pH 8.0, 1 mM EDTA). 16S and 23S ribosomal RNA (rRNA) were removed using the MICROBExpress Bacterial mRNA Enrichment kit (Life Technologies, Waltham, MA, USA). rRNA-depleted RNA samples were then resuspended in Rnase-free water and quantitated by a fluorescence-based Qubit RNA HS Assay kit (ThermoFisher Scientific) and fragmented for 4 min at 94 °C to be used as inputs for RNA library preparation. Illumina-compatible RNA libraries were prepared using a KAPA RNA HyperPrep Kit (KAPA Biosystems, Inc. Wilmington, MA, USA). The quality of the library and the size of the insert were determined by Agilent DNA High Sensitivity DNA chips; the Qubit dsDNA HS Assay (Thermo Fisher) was used to quantify these libraries. Purified libraries were clustered and sequenced on an Illumina HiSeq1500 in a 2 × 50 paired-end Rapid Run at the Marshall University Genomics Core Facility.

Sequencing reads were trimmed to remove Illumina adapter sequences and low-confidence base calls using Trimmomatic version 0.32 [38]. The quality of the resulting trimmed reads was verified using FastQC version 0.11.7 [39]. Counts of reads mapping to known genes for each sample were carried out using Salmon version 0.12 [40], with transcript sequences ASM924v1 (*Francisella tularensis* subsp. *holarctica* LVS) from GenBank as the reference transcripts. Resulting quantifications were imported into the R/Bioconductor package DESeq2 [41], and genes whose expression was altered by dillapiole treatment were identified using a false discovery rate threshold of 10%.

### 4.11. Western Blotting

Stationary-phase cultures of *F. tularensis* LVS were normalized to the lowest OD_600_ and mixed 1:1 with 2X Laemmli Buffer + 2.5% 2-Mercaptoethanol. Samples were boiled at 95 °C for 10 min. Subsequently, samples were loaded onto 4–15% polyacrylamide gels (IglC and IglA) or 12% polyacrylamide gels (LpnA) and run at 150 V for 45 min using Tris-Glycine-SDS running buffer. Proteins were transferred onto 0.45 μm nitrocellulose paper at 250 mA (100 V for 20 min) using Tris-Glycine Transfer Buffer (25 mM Tris, 192 mM Glycine, 20% (*v*/*v*) methanol). Blocking was carried out by treating the nitrocellulose membrane with PBS containing 0.02% sodium azide, 0.5% bovine serum albumin, 0.5% casein, and 100 mg/L Phenol Red for 30–60 min. Afterwards, blots were treated with primary antibodies (mouse anti-LpnA, mouse anti-IglC, and rabbit anti-IglA [both from BEI resources], 1:1000 dilution in killer filler) and incubated overnight with shaking at room temperature. Blots were then washed three times using room-temperature PBS. The washed blots were then treated with secondary antibodies (goat anti-rabbit IgG and rabbit anti-mouse, 1:2500 dilution in killer filler) incubated at room temperature for one hour. Following secondary antibody treatment, blots were washed twice with PBS followed by one wash with Tris Buffer. Blots were developed using Naphthol AS-MX phosphate (1 mg/mL) and Fast Red TR salt (2 mg/mL) dissolved in Tris Buffer. Once red bands appeared, nitrocellulose paper was washed thoroughly with tap water to prevent overdevelopment. ImageJ was used to quantify the intensity of each band.

### 4.12. Statistical Analyses

All statistical calculations were conducted using GraphPad Prism software.

## 5. Patent

The use of dillapiole and derivatives as a novel anti-virulence therapeutic was patented on 28 October 2024. US Patent Number: US-12109187-B2

## Figures and Tables

**Table 1:** Sources of extracts with novel antimicrobial activity.

Extract ID	Scientific Name	Common Name	Plant Part
NPX 107 B4	*Epilobium angustifolium* L.	Willow herb	Stems
NPX 107 H4	*Chamaebatia foliolosa* Benth.	Mountain misery	Leaves
NPX 107 A10	*Solidago spectabilis* A. Gray	Nevada goldenrod	Flowers
NPX 108 B7	*Agastache parvifolia* Eastw.	Small leaf giant hyssop	Flowers
NPX 131 B1	*Foeniculum vulgare* Mill.	Fennel, Hinojo	Roots
NPX 131 B10	*Styrax officinalis* L.	Storax tree	Stems
NPX 131 F10	*Epilobium angustifolium* L.	White-bark pine	Stems
NPX 133 B6	*Chamaebatia foliolosa* Benth.	Hercules’ club	Stems
NPX 133 B10	*Solidago spectabilis* A. Gray	Ocotillo	Stems

**Table 2:** *F. tularensis LVS* genes controlled by MglA and SspA, which exhibit significantly decreased expression following treatment with dillapiole. FPI genes are highlighted in dark gray. Genes controlled by SspA located outside the FPI are highlighted in light gray.

Locus Tag	Gene Name	Fold Downregulated
FTL_0026	3-hydroxyisobutyrate dehydrogenase	2.14
FTL_0113	Intracellular growth locus, subunit C, *iglC*	3.08
FTL_0115	Pathogenicity determinant protein E, *pdpE*	2.42
FTL_0116	Pathogenicity determinant protein C, *pdpE*	2.18
FTL_0118	Intracellular growth locus, subunit I, *iglI*	2.48
FTL_0119	Type VI secretion system protein, *dotU*	2.65
FTL_0120	Intracellular growth locus, subunit H, *iglH*	2.08
FTL_0121	Intracellular growth locus, subunit G, *iglG*	1.88
FTL_0122	Intracellular growth locus, subunit F, *iglF*	1.78
FTL_0123	Valine–glycine repeat protein G, *vgrG*	1.91
FTL_0124	Intracellular growth locus, subunit E, *iglE*	3.30
FTL_0125	Pathogenicity determinant protein B, *pdpB*	1.94
FTL_0126	Pathogenicity determinant protein A, *pdpA*	2.20
FTL_0499	S-adenosylmethionine decarboxylase	1.74
FTL_0673	Pantoate-beta-alanine ligase	1.69
FTL_0674	3-methyl-2-oxobutanoate hydroxymethyltransferase	1.64
FTL_1606	Stringent starvation protein A, *sspA*	1.30

## Data Availability

Raw and processed RNA-Seq data were deposited in the Gene Expression Omnibus maintained by the National Center for Biotechnology Information at the National Library of Medicine. Data are available via accession numbers GSE30611 (sequencing of *F. tularensis*) and GSE306199 (THP-1 cells).

## Supplementary Materials

The following supporting information can be downloaded at https://www.mdpi.com/article/10.3390/molecules30193995/s1, NMR spectroscopic and spectrometric data, Figure S1. Proton NMR of dillapiole in C_6_D_6_; Figure S2. Carbon NMR of dillapiole in C_6_D_6_; Figure S3. COSY experiment of dillapiole in C_6_D_6_; Figure S4. HSQC experiment of dillapiole in C_6_D_6_; Figure S5. HMBC experiment of dillapiole in C_6_D_6_; Figure S6. Proton NMR of dillapiole in CDCl_3_; Figure S7. Proton NMR of dillapiole in CDCl_3_; Figure S8. Low-resolution ESI of dillapiole; Figure S9. Ingenuity Pathway Analysis (IPA) revealed that 45 pathways were affected by dillapiole treatment; Figure S10. Dillapiole inhibits replication of *F. tularensis* in RAW 264.7 mouse macrophages; Figure S11. Dillapiole dampens TNF-α production by RAW 264.7 mouse macrophages; Figure S12. ELISA confirms that TNF-alpha production decreases in response to dillapiole in RAW 264.7 cells.

## Abbreviations

The following abbreviations are used in this manuscript:

CFU	Colony-Forming Units
CDC	Centers for Disease Control and Prevention
FPI	*Francisella* Pathogenicity Island
T6SS	Type VI Secretion System
FDA	Food and Drug Administration
TLC	Thin-Layer Chromatography
NMR	Nuclear Magnetic Resonance
MIC	Minimum Inhibitory Concentration
LVS	Live Vaccine Strain
