# Supplementary material for: Dillapiole Dampens the Expression of the Major Virulence Genes of Francisella tularensis
Source: Molecules. 2025 Oct 6;30(19):3995. doi: 10.3390/molecules30193995 (PMC12526145; doi:10.3390/molecules30193995)
Supplement: Supplementary file 1 [file molecules-30-03995-s001.zip › molecules-3861742-supplementary.pdf]

Dillapiole dampens the expression of the major virulence genes of *Francisella tularensis*

Elliot M. Collins[1], Anthony Sako[1], Kristen Sikorsky[1], James Denvir[2], Jun Fan[3], Donald A. Primerano[2], Deanna M. Schmitt[1], Stuart Cantlay[1], Roger Seeber[1], Francisco León[4], & Joseph Horzempa[1*]

[1] Department of Biological Sciences, West Liberty University, West Liberty, WV, USA
[2] Department of Biomedical Sciences, Joan C. Edwards School of Medicine, Marshall University, Huntington, WV, USA
[3] Texas A&M Institute for Genome Sciences and Societies, Texas A&M University, College Station, TX, USA
[4] Department of Drug Discovery and Biomedical Sciences, College of Pharmacy, University of South Carolina, Columbia, SC, USA
* Correspondence: joseph.horzempa@westliberty.edu

TABLE OF CONTENT

N_0		Page
1	Spectroscopic and spectrometric data for dillapiole	3
2	**Figure 1S**. Proton NMR of dillapiole in C_6D_6	4
3	**Figure 2S**. Carbon NMR of dillapiole in C_6D_6	5
4	**Figure 3S**. COSY experiment of dillapiole in C_6D_6	6
5	**Figure 4S**. HSQC experiment of dillapiole in C_6D_6	7
6	**Figure 5S**. HMBC experiment of dillapiole in C_6D_6	8
7	**Figure 6S**. Proton NMR of dillapiole in $CDCl_3$	9
8	**Figure 7S**. Proton NMR of dillapiole in $CDCl_3$	10
9	**Figure 8S**. Low resolution ESI of dillapiole	11
10	**Figure 9S.** Ingenuity Pathway Analysis (IPA) revealed that 45 pathways were affected by dillapiole treatment.	12
11	**Figure 10S.** Dillapiole inhibits replication of *F. tularensis* in RAW 264.7 mouse macrophages.	13
12	**Figure 11S**. Dillapiole dampens TNF-α production by RAW 264.7 mouse macrophages.	14
13	**Figure 12S.** ELISA confirms that TNF-α production decreases in response to dillapiole in RAW 264.7 cells.	15

Experimental data.

Dillapiole: ^{1}H NMR (500 MHz, C_6D_6) δ 6.41 (s, 1H), 5.91 (ddt, *J* = 16.7, 10.0, 6.6 Hz, 1H), 5.25 (s, 2H), 5.02 (dq, *J* = 17.0, 1.7 Hz, 1H), 4.98 (dq, *J* = 10.0, 1.4 Hz, 1H), 3.76 (s, 3H), 3.64 (s, 3H), 3.32 (d, *J* = 6.6 Hz, 2H). ^{13}C NMR (126 MHz, C_6D_6) δ 145.3, 145.1, 138.4, 137.9, 136.8, 126.4, 115.5, 103.3, 101.0, 61.0, 59.7, 34.4; ^{1}H NMR (400 MHz, $CDCl_3$) δ 6.35 (s, 1H), 5.97 – 5.86 (m, 3H), 5.09 – 5.04 (m, 1H), 5.03 (t, *J* = 1.4 Hz, 1H), 4.01 (s, 3H), 3.76 (s, 3H), 3.31 (dt, *J* = 6.5, 1.5 Hz, 2H). ^{13}C NMR (101 MHz, $CDCl_3$) δ 144.7, 144.5, 137.8, 137.5, 136.1, 126.2, 115.7, 102.9, 101.2, 61.4, 60.1, 34.1. ESI-MS: m/z 223 $[M+H]^{+}$.

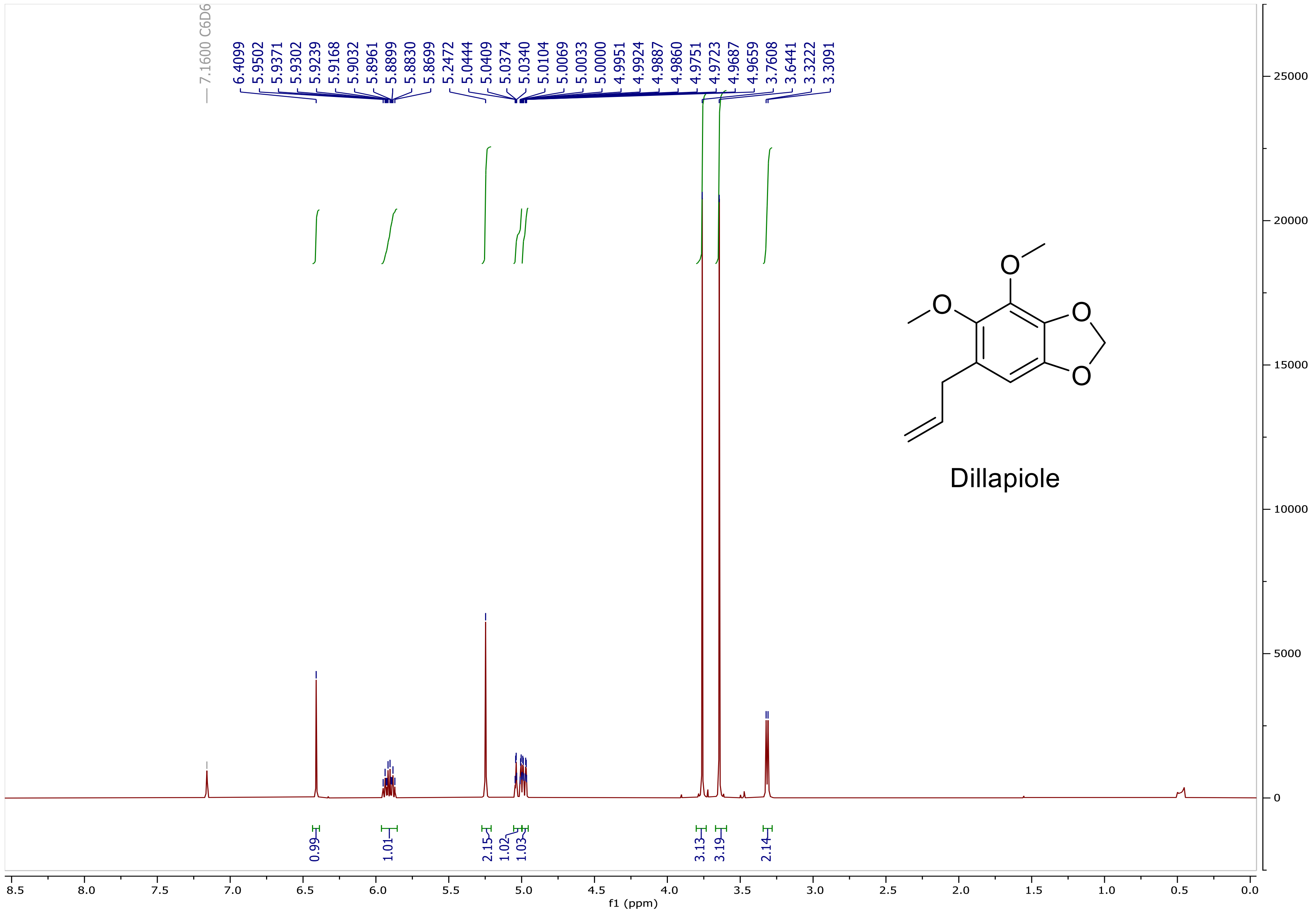

Figure 1S. Proton NMR of dillapiole in C_6D_6**.**

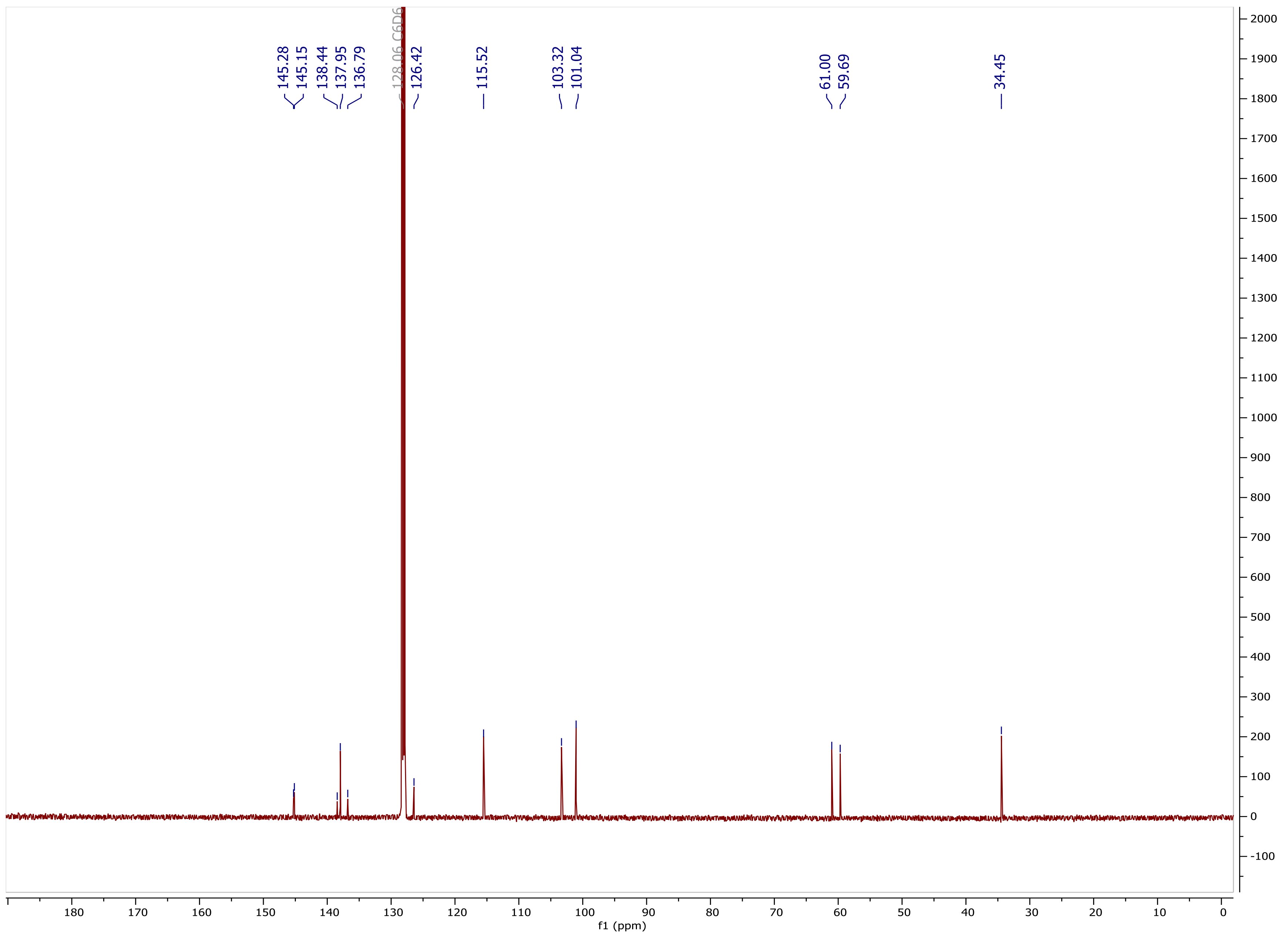

Figure 2S. Carbon NMR of dillapiole in C_6D_6**.**

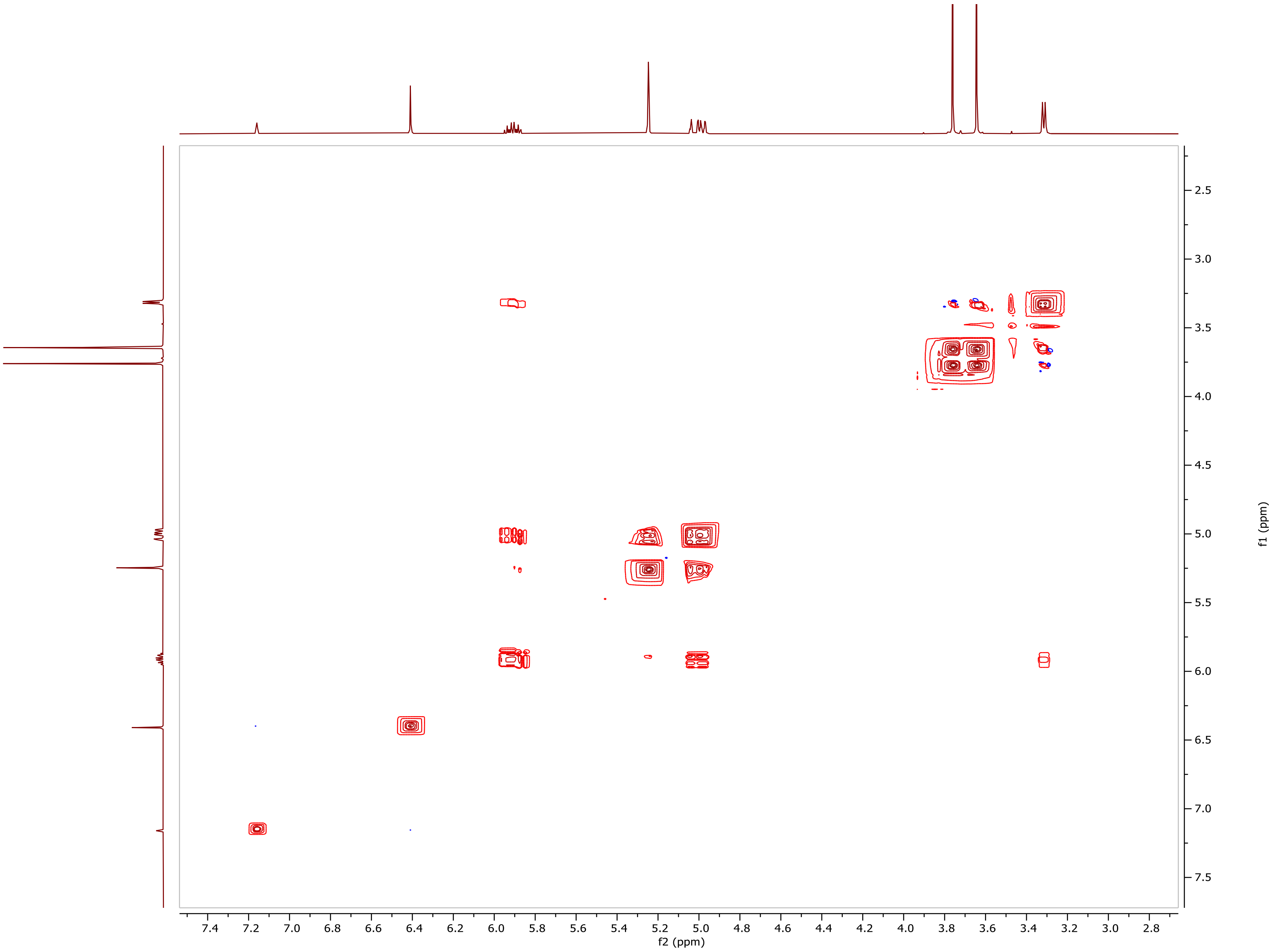

Figure 3S. COSY experiment of dillapiole in C_6D_6.

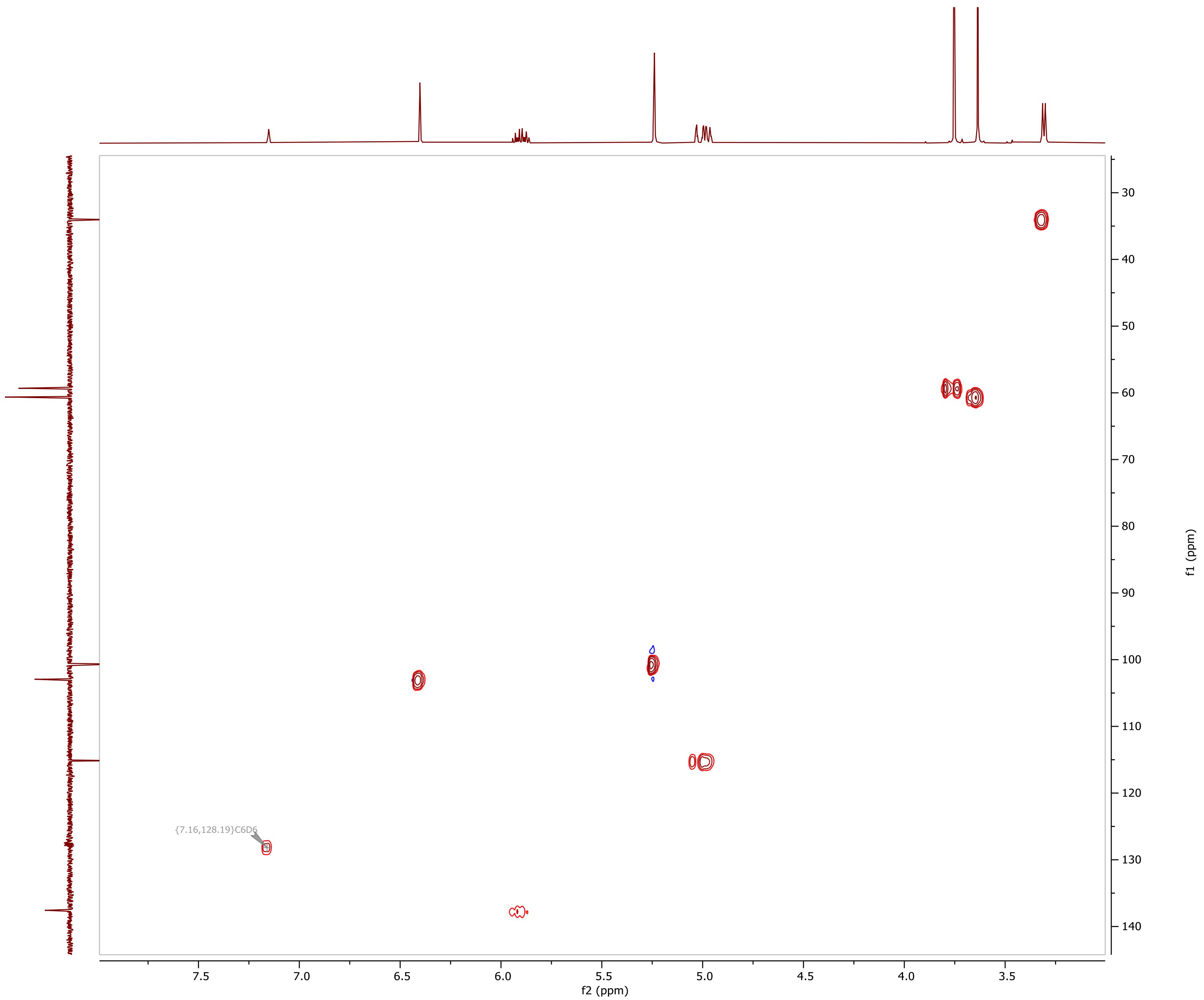

Figure 4S. HSQC experiment of dillapiole in C_6D_6.

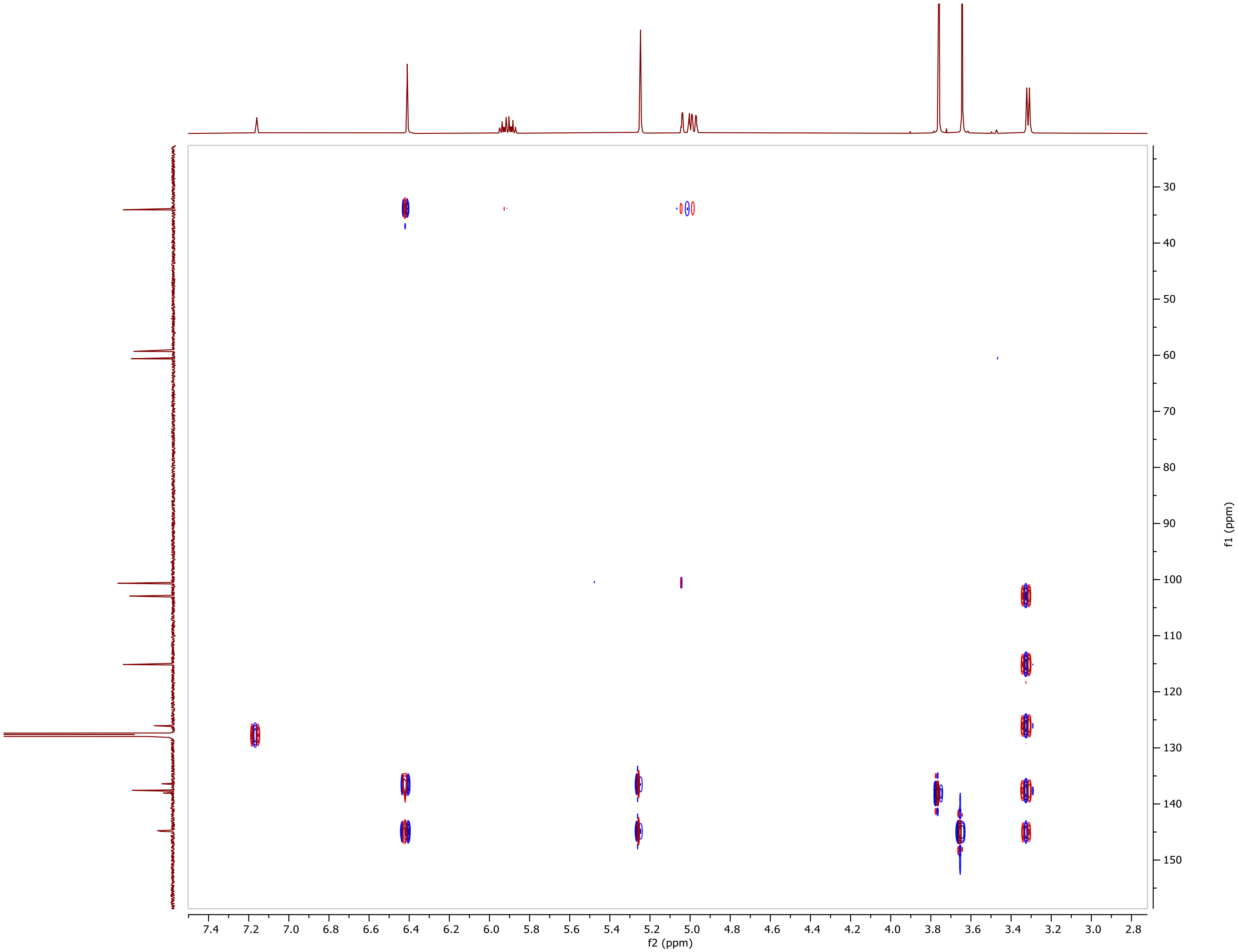

Figure 5S. HMBC experiment of dillapiole in C_6D_6**.**

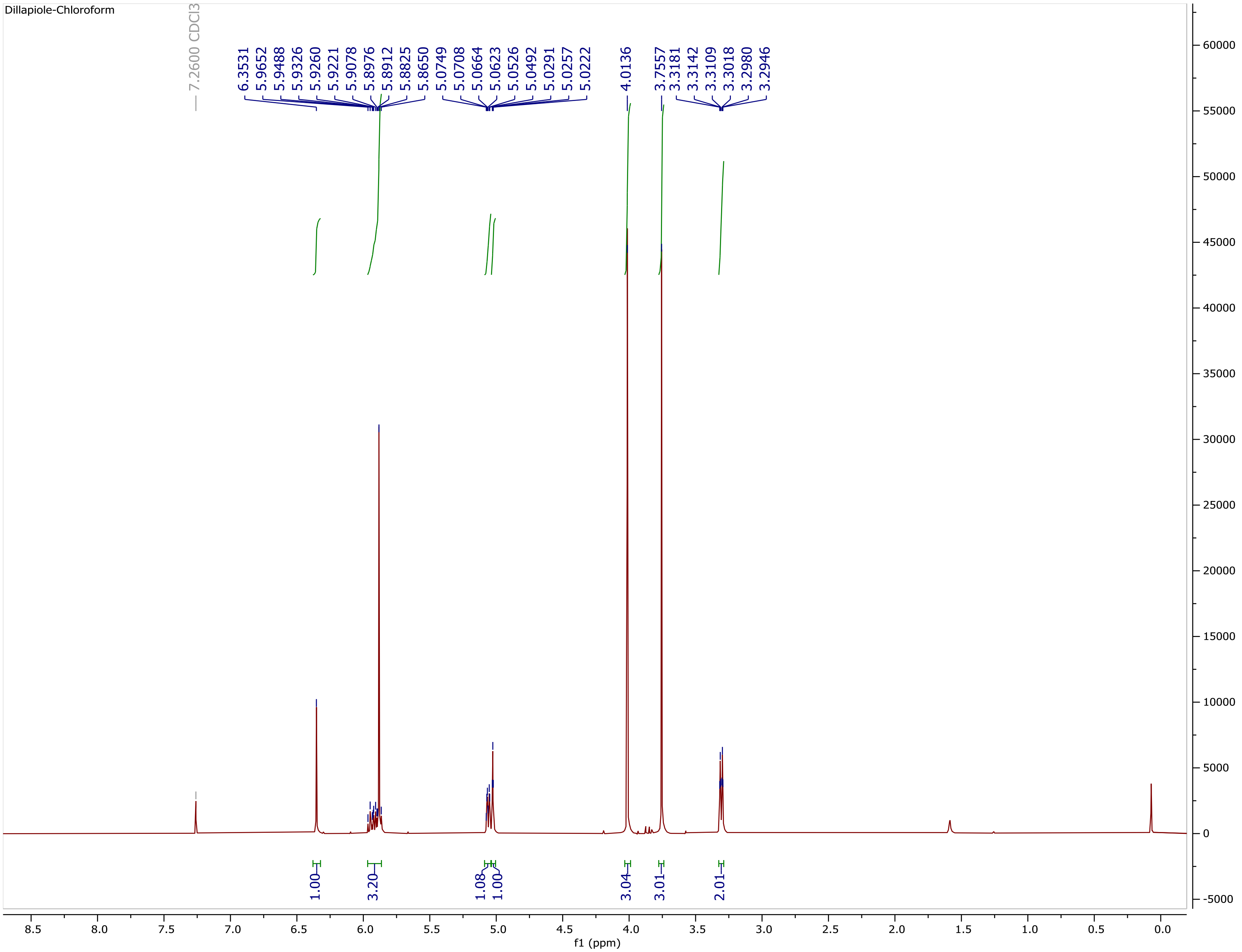

Figure 6S. Proton NMR of dillapiole in $CDCl_3$

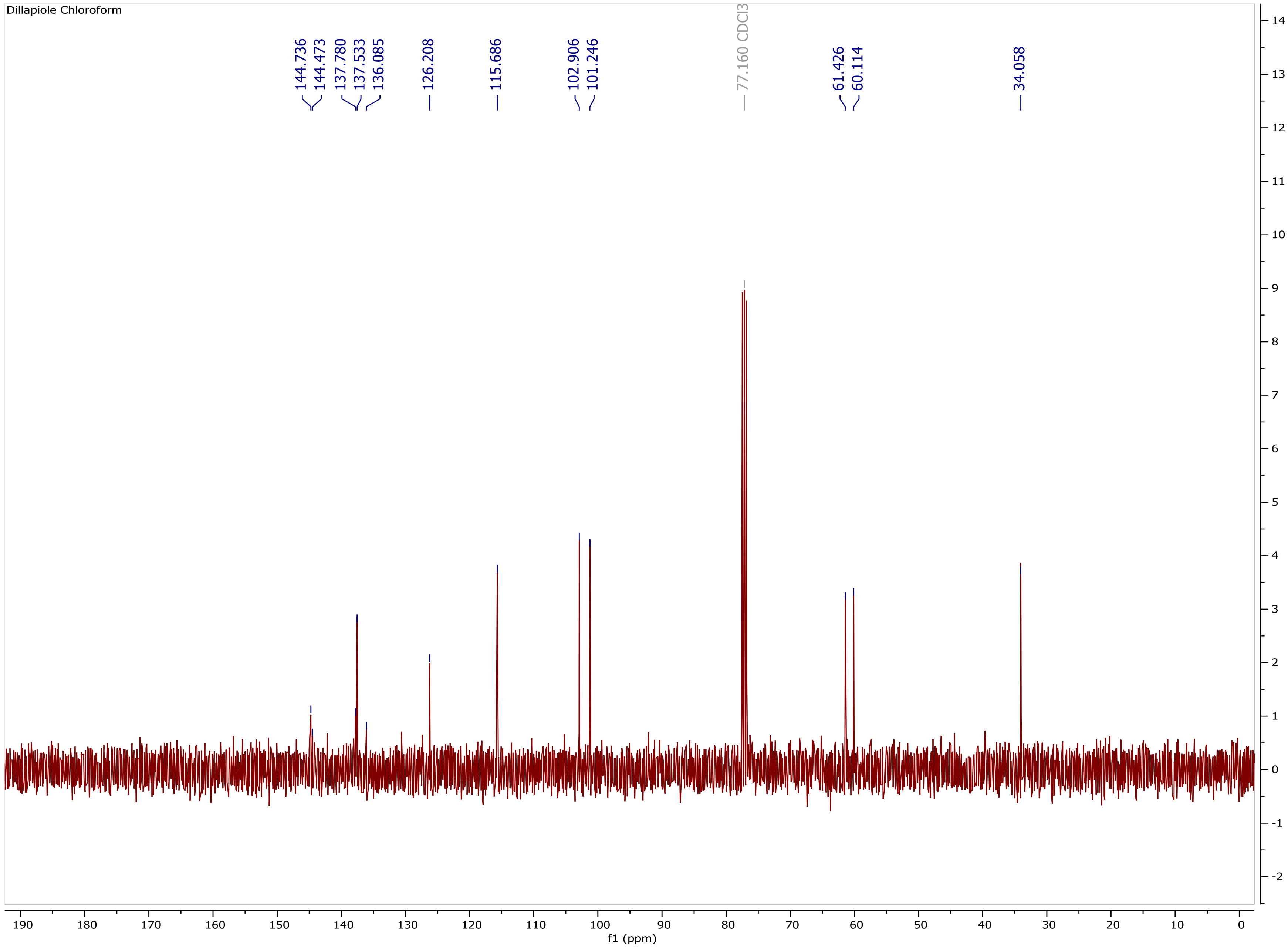

Figure 7S. Proton NMR of dillapiole in $CDCl_3$

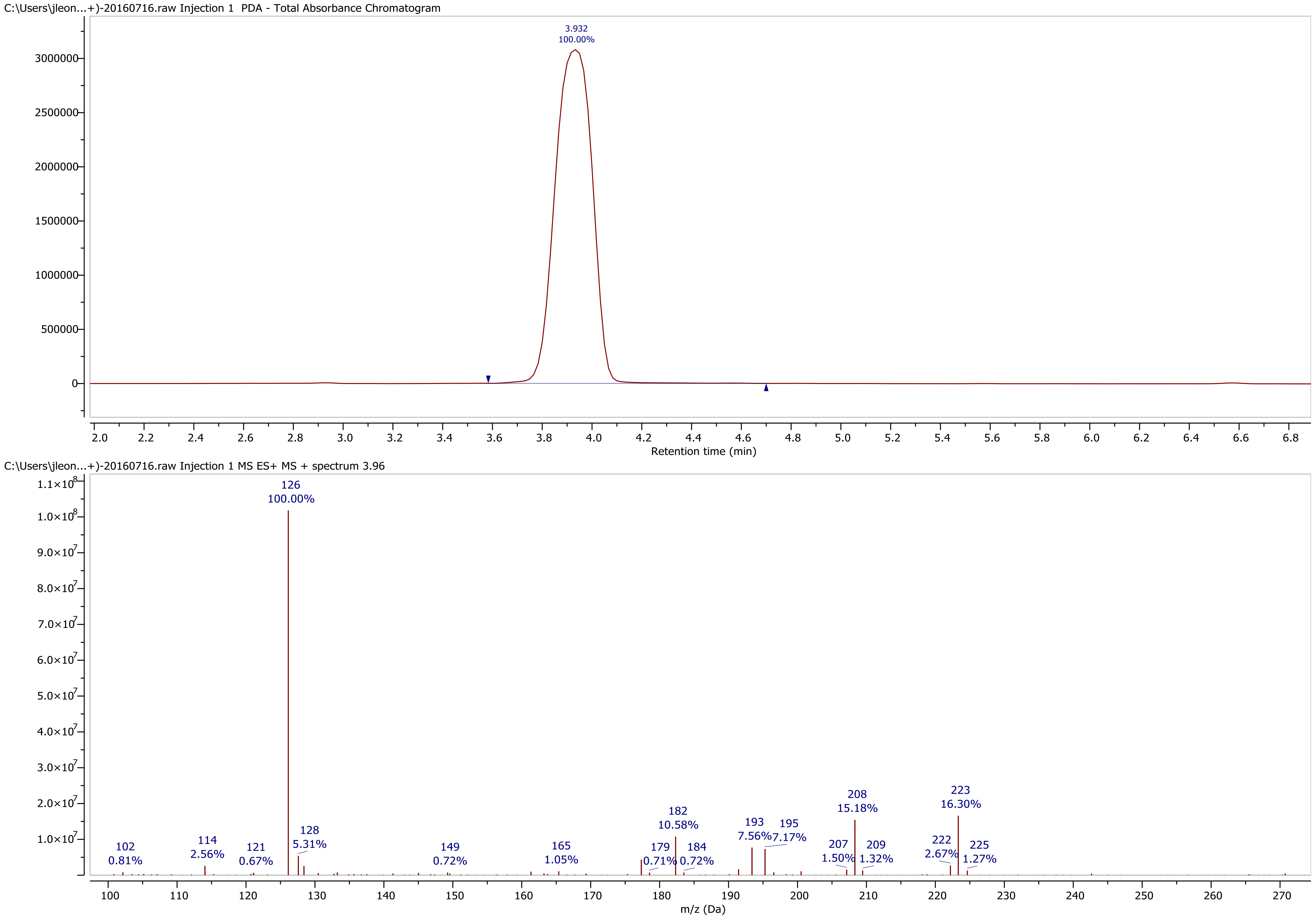

Figure 8S. Low resolution ESI of dillapiole**.**

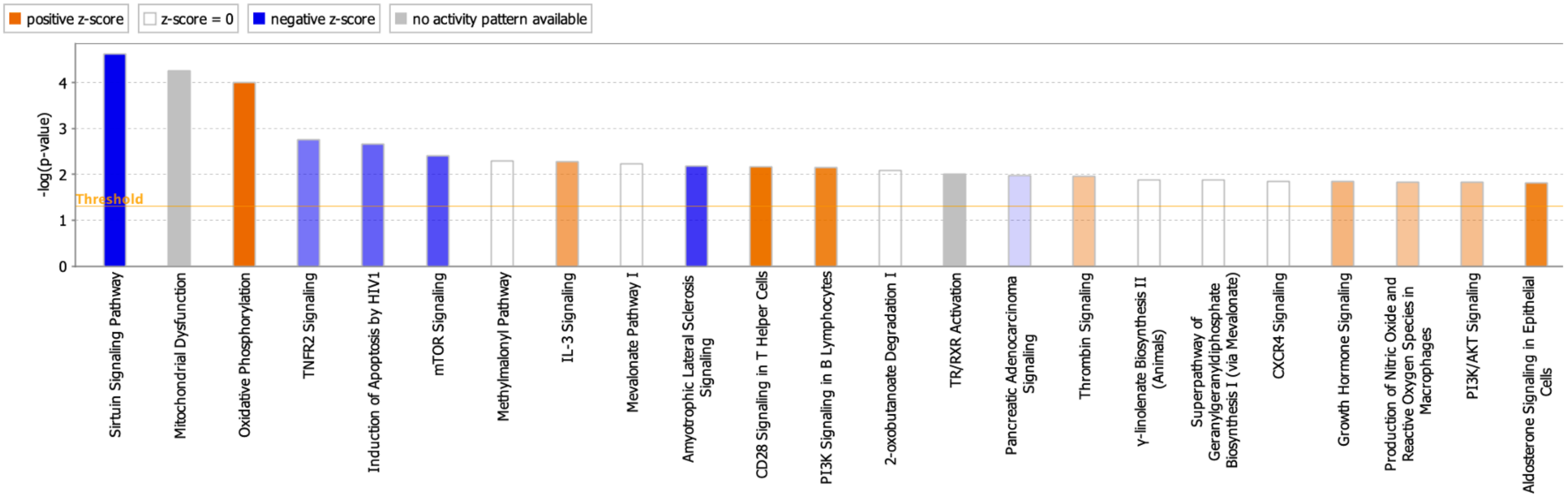

Figure 9S. Ingenuity Pathway Analysis (IPA) revealed that 45 pathways were affected by dillapiole treatment. The top 23 are shown. Upregulated pathways are shown in orange, and downregulated are shown in blue.

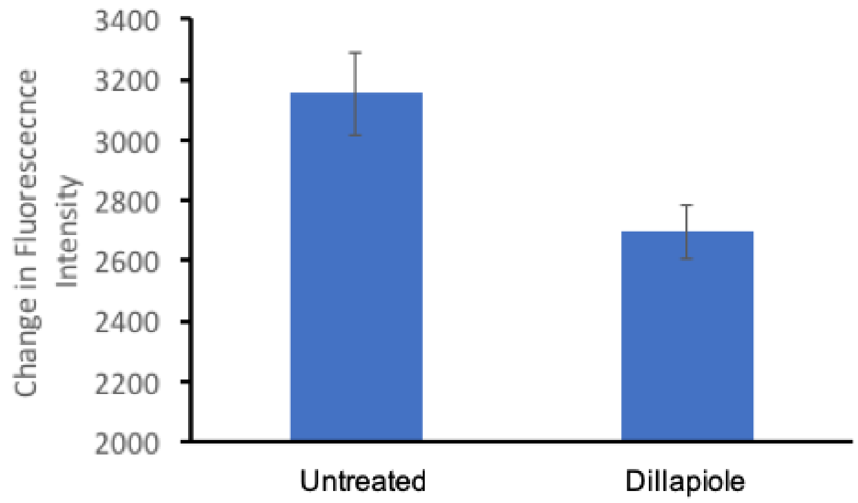

Figure 10S. Dillapiole inhibits replication of *F. tularensis* in RAW 264.7 mouse macrophages. RAW 264.7 cells were infected with LVS / pTC3D. Cells were left untreated or were treated with dillapiole. Differences in fluorescence intensity over a 48h infection were measured (mean change in fluorescence ± SEM shown).

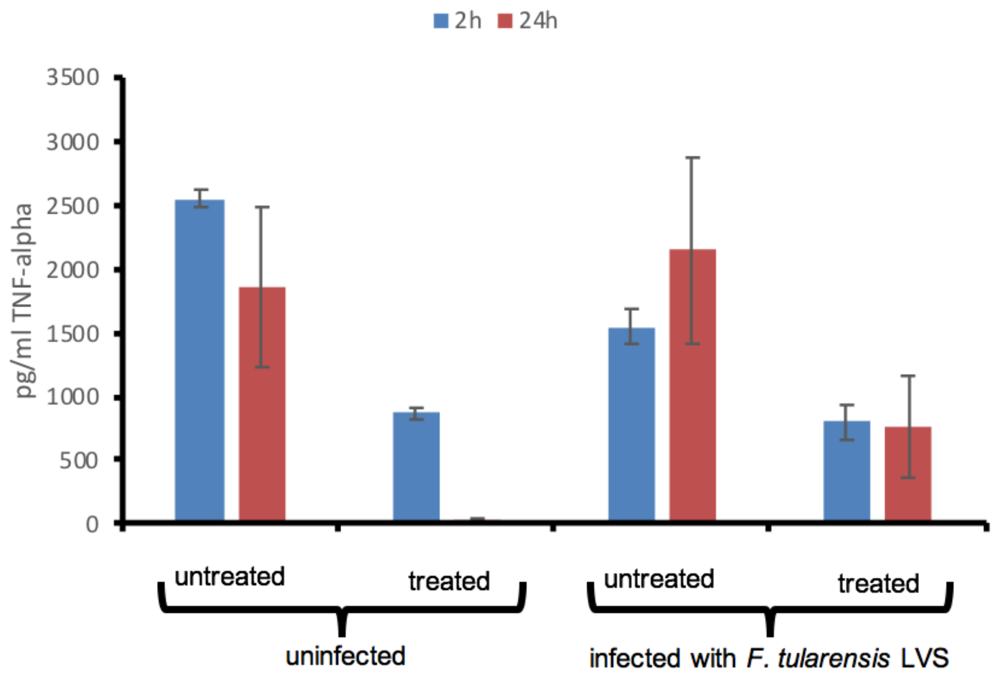

Figure 11S. Dillapiole dampens TNF-α production by RAW 264.7 mouse macrophages. RAW 264.7 cells (either infected with *F. tularensis* LVS or left uninfected) were treated with dillapiole or left untreated. At the time points following dillapiole treatment indicated, the culture supernatants were subjected to a Bio-Rad multiplex cytokine / chemokine array. Data shown are mean TNF-α ± SD from representative experiment. An independent biological replicate experiment showed similar results (data not shown).

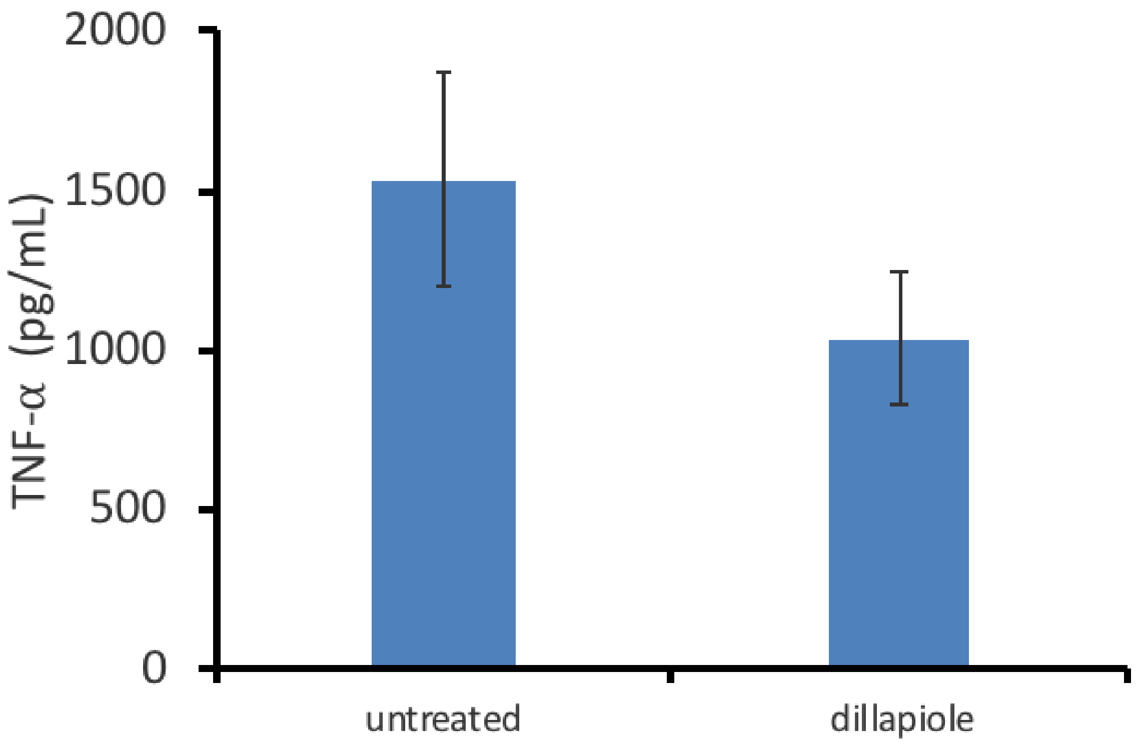

Figure 12S. ELISA confirms that TNF-α production decreases in response to dillapiole in RAW 264.7 cells. RAW 264.7 macrophages were infected with *F. tularensis* LVS and treated with dillapiole or were left untreated. After 24h, culture supernatants were subjected to ELISA (R&D systems). Data shown are mean TNF-α concentration ±SD.
